# Supplementary material for: Polyethylene glycol grafted with carboxylated graphene oxide as a novel interface modifier for polylactic acid/graphene nanocomposites
Source: R Soc Open Sci. 2020 Jul 22;7(7):192154. doi: 10.1098/rsos.192154 (PMC7428252; doi:10.1098/rsos.192154)
Supplement: Supplementary Material [file rsos192154supp2.docx]

**Supporting Information**

**Polyethylene glycol grafted with carboxylated graphene oxide as a novel interface modifier for polylactic acid/graphene nanocomposites**

Mingjun Niu ^1^, Hao Wang ^1^, Jing Li ^1^, Hongyan Chen ^1^, Lin li ^1^, Huige Yang ^1^, Xuying Liu ^1^, Zhihao Chen ^2^, Hongzhi Liu^3^ and Jinzhou Chen ^1^

^1^ School of Materials Science and Engineering, Zhengzhou University, Zhengzhou 450001, China.

^2^ Zhengzhou Tobacco Research Institute of CNTC, Zhengzhou 450001, Henan, China. ^3^ School of Chemical and Biological Engineering, NingboTech University, No. 1 Xuefu Road, Ningbo 315100, P. R. China

Corresponding authors.

To whom the correspondence should be addressed: E-mail: yanghg@zzu.edu.cn (Prof. Huige Yang), chenzh@ztri.com.cn(Dr. Zhihao Chen) and cjz@zzu.edu.cn (Prof. Jinzhou Chen).


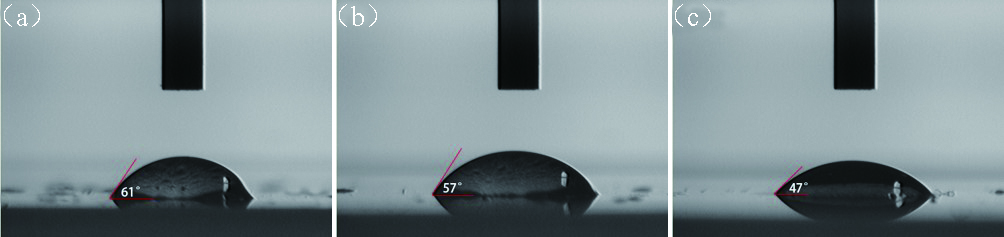


**Fig. S1** Water contact angle measurements of : (a) GO and (b) GC25 and (c) GC50 films coated evenly on glasses

As can be seen from Fig. S1, the water contact angle of GO is 61°, which indicates that GO exhibits hydrophilic properties. When carboxylation occurs, the contact angle decreases to 57° and 47°, respectively. This is due to the fact that the carboxyl group is more hydrophilic than the hydroxyl group.





**Fig. S2** DSC heating curves of the PLA/GO-g-PEG nanocomposites at a scan rate of 10 °C min^-1^

The samples of pure PLA and PLA/GO-g-PEG nanocomposites were tested by DSC, and were heated from room temperature to 190 °C at a scanning rate of 10 °C min^-1^. The corresponding parameters obtained from the curve are listed in the Table S1. The crystallinity(X_c_) of PLA can be calculated by comparing the melting enthalpy(ΔH_m_) of PLA with the theoretical melting enthalpy(93 J g^-1^). From figure S2, it can be concluded that the addition of GO-g-PEG reduces the Tg and Tcc of PLA. With the increase of GO-g-PEG content, Tg and Tcc show the trend of first decreasing and then rising. At the same time, the addition of PLA can improve the X_c_ of PLA. When the content of PLA is 0.4%, the maximum crystallinity of PLA is 37.8%.

Table S1. DSC results of pure PLA and PLA/GO-g-PEG nanocomposites samples

| Samples | T_g_(°C) | T_cc_(°C) | ΔH_m_(°C) | X_C_(%) |
| --- | --- | --- | --- | --- |
| PLA | 62.3 | 122.3 | 15.1 | 16.2 |
| PLA/GO-g-PEG 0.1 | 60 | 106.5 | 30.5 | 32.8 |
| PLA/GOg-PEG 0.2 | 59.4 | 105.1 | 32.0 | 34.4 |
| PLA/GO-g-PEG 0.3 | 58.7 | 103.5 | 33.7 | 36.3 |
| PLA/GO-g-PEG 0.4 | 56.6 | 102.5 | 35.2 | 37.8 |
| PLA/GO-g-PEG 0.5 | 57.7 | 103.2 | 34.1 | 36.7 |





**Fig. S3** Typical nominal stress-strain curves for pure PLA and PLA/GO-g-PEG nanocomposites with various loadings

Table S2. Tensile Properties of pure PLA and PLA/GO-g-PEG nanocomposites with various loadings

| Samples | Tensile Strength(MPa) | Elongation at Break(%) | Modulus(GPa) | Tensile toughness(MJ m^-3^) |
| --- | --- | --- | --- | --- |
| PLA | 63 | 4 | 2.1 | 20.1 |
| PLA/GO-g-PEG 0.1 | 62 | 10 | 1.9 | 54.9 |
| PLA/GO-g-PEG 0.2 | 61 | 16 | 1.8 | 88.2 |
| PLA/GO-g-PEG 0.3 | 61 | 18 | 1.6 | 101.4 |
| PLA/GO-g-PEG 0.4 | 60 | 22 | 1.8 | 120.8 |
| PLA/GO-g-PEG 0.5 | 59 | 14 | 1.7 | 74.6 |


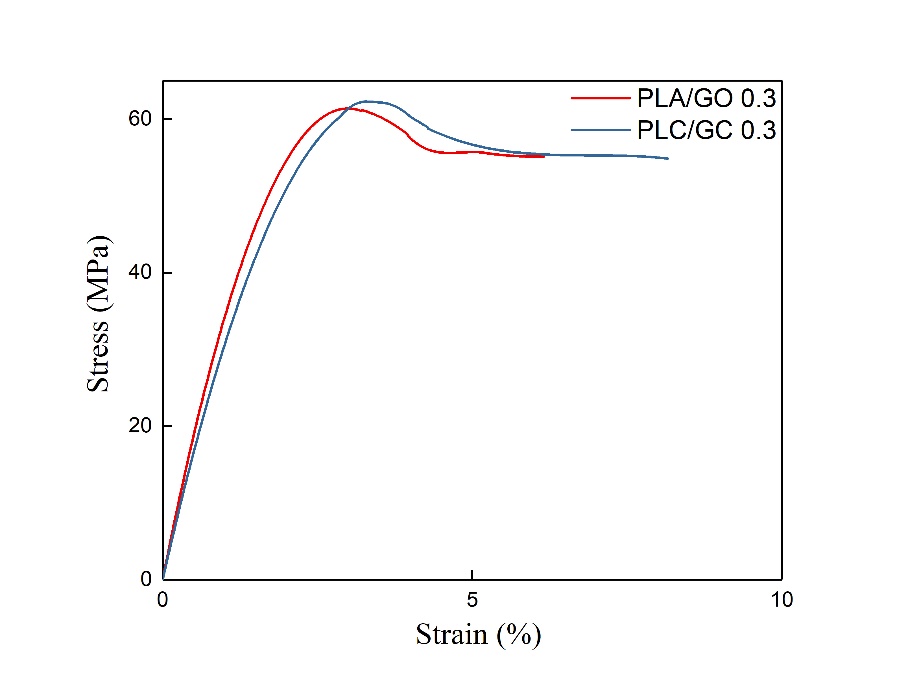


**Fig. S4** Typical nominal stress-strain curves for PLA/GO 0.3 and PLA/GC 0.3

Table S3. Tensile Properties of PLA/GO 0.3 and PLA/GC 0.3

| Samples | Tensile Strength(MPa) | Elongation at Break(%) | Modulus(GPa) | Tensile toughness  (MJ m^-3^) |
| --- | --- | --- | --- | --- |
| PLA/GO 0.3 | 61 | 6 | 1.89 | 30.3 |
| PLA/GC 0.3 | 62 | 8 | 1.86 | 40.9 |

As shown in Fig S3, with the increase of GO-g-PEG content, the tensile strength of PLA decreases, and the elongation at break continued to increase. This phenomenon continued until the addition amount is 0.4%, at which time the elongation at break reached the maximum of 22%, tensile strength and modulus decreases by around 3.8 and 14%, respectively. When the content of GO-g-PEG is 0.5%, all mechanical properties decline, because too many nanofillers will cause agglomeration in the process of processing. It also can be seen that the addition of GO-g-PEG increases the elongation at break of PLA, but the rate of increase and the maximum increase are not as good as that of GC-g-PEG at the same content.

Similarly, the PLA/GO and PLA/GC nanocomposites are shown in Fig. S4 and the tensile properties are presented in Table S3. Both PLA/GO 0.3 and PLA/GC 0.3 are low-ductility, which can be rationalized in terms of the incompatibility between the PLA and nanofillers.


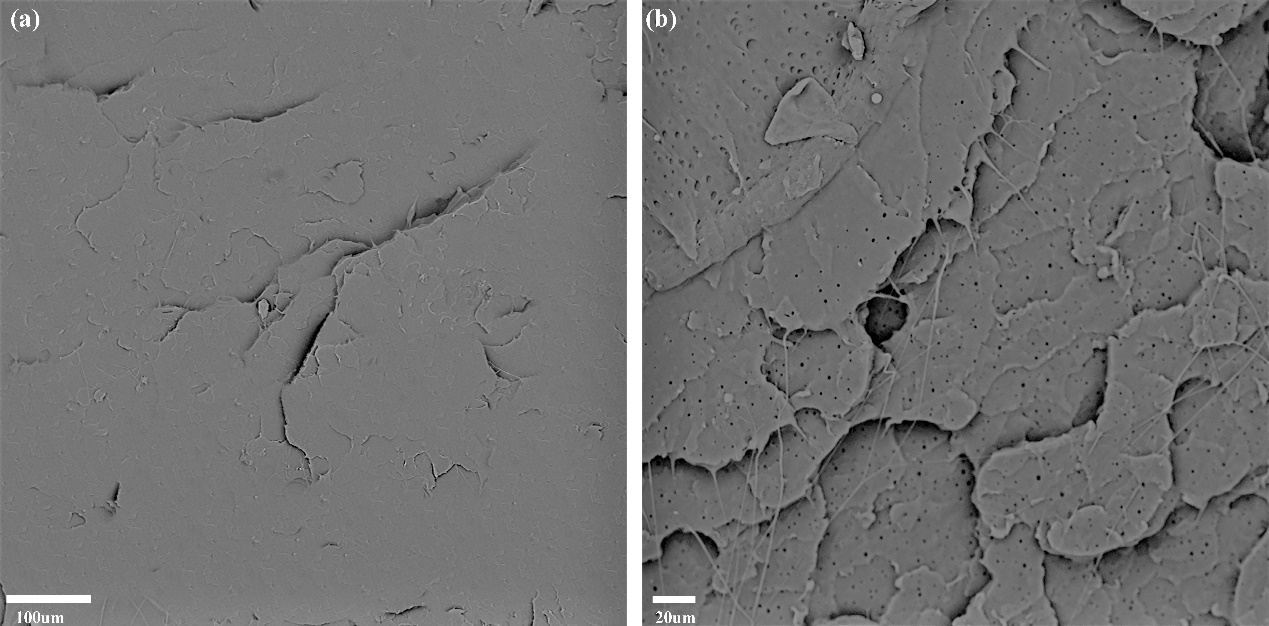


**Fig. S5** The SEM micrographs (low magnification) of the nanocomposites fracture surfaces: (a) pure PLA and (b) PLA/GC-g-PEG 0.3

Fig. S5 shows the SEM graphs of the tensile fracture surfaces of pure PLA and PLA/GC-g-PEG 0.3 at low magnification. It can be seen from Fig. S5(a) that pure PLA is a relatively smooth section. After 0.3% GC-g-PEG was added, many holes appear on the fracture surface.
